# Therapeutic effect and tolerability of gelatin sponge particle-mediated chemoembolization for colorectal liver metastases: a retrospective study

**DOI:** 10.1186/1477-7819-11-222

**Published:** 2013-09-09

**Authors:** Chuang Li, Yuewei Zhang, Jun Zhou, Guangsheng Zhao, Shunxiong Tang

**Affiliations:** 1 Department of Interventional Radiology, Affiliated Zhongshan Hospital of Dalian University, 6 Jiefang Street, Zhongshan District, Dalian 116001, China

**Keywords:** Colorectal cancer, Liver metastases, GSP, Chemoembolization

## Abstract

**Background:**

Colorectal cancer (CRC) is one of the most common cancers worldwide. The majority (approximately 60%) of patients with CRC will eventually develop liver metastases, which remain the most common cause of mortality in these patients. This study aimed to evaluate the therapeutic effect and tolerability of gelatin sponge particle (GSP)-mediated chemoembolization in the treatment of colorectal liver metastases after systemic chemotherapy failure.

**Methods:**

This was a single-center retrospective study of 15 patients with colorectal liver metastases, who underwent GSP-mediated chemoembolization with 50 mg of lobaplatin during the period December 2009 to December 2010 in the Department of Interventional Radiology, Affiliated Zhongshan Hospital of Dalian University. Clinical data were retrieved, and the therapeutic effect and tolerability of the treatment were evaluated.

**Results:**

All 15 patients with colorectal liver metastases completed the GSP-mediated chemoembolization. The therapeutic effect and tolerability were evaluated 3 months after the initial procedure. The tumor lesions in all patients showed various levels of necrosis and shrinkage. According to the Response Evaluation Criteria in Solid Tumors (RECIST), one patient achieved complete response (CR), eleven patients achieved partial response (PR), and three patients achieved stable disease (SD). The overall response rate (CR + PR) was 80%.

**Conclusions:**

GSP-mediated chemoembolization is well tolerated and has a good short-term response rate (80%) in the treatment of colorectal liver metastases after systemic chemotherapy failure. Collectively, further study of the long-term effect of GSP-mediated chemoembolization in colorectal liver metastasis in a large cohort is warranted.

## Background

The incidence rate of colorectal cancer ranks second for malignancies worldwide, and colorectal cancer (CRC) is the third leading cause of cancer death in developing countries [1]. Liver metastasis is a common clinical symptom of CRC, and is one of the main factors that threatens patients’ lives and affects their survival [2,3]. It was reported that liver metastases occurred in 60 to 70% of patients with CRC, 20 to 50% of whom already had liver metastases when the clinical diagnosis was confirmed [4,5]. For patients with liver metastases, surgical resection is still the primary therapeutic method for prolonging survival [6-9]. Unfortunately, only 15 to 30% of patients undergo surgery due to surgical contraindications and unwillingness of patients [10]. For patients with unresectable disease, it is essential to identify therapies that can provide a high response rate and prolong survival.

Transcatheter arterial chemoembolization (TACE) is an intra-arterial therapy that restricts the arterial vascular supply of liver metastases. Chemotherapeutic agents combined with embolization particles result in high doses of drugs directly to the liver, and selectively cause ischemic damage to the hepatic tumors. Recently, TACE has become increasingly important in comprehensive cancer treatment for colorectal liver metastases [11]. Gelatin sponge particles (GSPs) provide an effective absorbable embolization agent. Compared with the conventional permanent embolization agent Lipiodol/Ethiodol (ethiodized oil), GSP has advantages such as absorbability, no mass effect, and complete embolism [12]. Additionally, the GSPs (350 to 560 μm in size) used in this study have an absorption time of 7 to 14 days and are more homogeneous than other absorbable embolization agents such as soluble starch microspheres. Because of these advantages, GSPs cause less damage to hepatic function, and complete embolization of the tumor-feeding arteries can be accomplished.

Although application of GSPs in the treatment of primary hepatocelluar carcinoma has been reported [13], there are very few clinical reports of GSPs being used in colorectal liver metastases. In this study, a retrospective analysis was conducted on 15 patients with colorectal liver metastases who underwent GSP-mediated chemoembolization. The therapeutic effect and tolerability were evaluated.

## Methods

### Study methods and eligibility criteria

This study was a single-center retrospective review of 15 patients with colorectal liver metastases who underwent GSP-mediated chemoembolization during the period December 2009 to December 2010 in our department. The aim of this study was to evaluate the therapeutic effect and tolerability of this treatment. All patients underwent radical surgical resection of the primary CRC. The intervals between the operation on the primary CRC and initiation of TACE ranged from 3 months to 2 years. At the time of considering GSP-mediated chemoembolization as a ‘salvage’ therapy, all patients had progressive stage IV disease after failure of systemic chemotherapy (5-fluorouracil (5-FU)/leucovorin/oxaliplatin (FOLFOX) and 5-FU/leucovorin/irinotecan (FOLFIRI)). Owing to patient choice (3 cases) and surgical contraindications, such as bilobar tumor distribution and involvement of major hepatic vascular structures (12 cases), these patients were deemed unresectable. All patients signed informed consent for the treatment. In accordance with institutional (Affiliated Zhongshan Hospital of Dalian University) ethical practice, alterative treatment options were provided with explanation of risks and benefits.

### Materials used

The GSPs (registration number, CFDA [2006] number 3770360; product standard, YZB/State 2518-77-2004 (Gelatin sponge microparticle embolization agents); Yilikang Pharmaceutical Co., Ltd. (Hangzhou, China) used ranged in size from 350 to 560 μm. The chemotherapy drug used was lobaplatin for injection (registration number, CFDA [2006] number H20080359; 50 mg each; Chang’an International Pharmaceutical Co., Ltd. (Hainan, China).

### Interventional treatment

Digital subtraction angiography (DSA), a procedure to identify tumor vasculature using radiation, was completed in the 15 patients before chemoembolization. The interventional treatment was performed using the Seldinger technique. A licensed surgeon from our department with 15 years of experience performed these procedures. Briefly, patients underwent percutaneous femoral artery catheterization and selective arteriography. After determining the source of the tumor-feeding arteries, the lesion numbers, and the staining intensity, the chemoembolization with 50 mg of lobaplatin and GSPs was performed on the arteries in the region of the tumor blood supply to attain complete arterial blockage. One month after the procedure, enhanced computed tomography (CT) of the upper abdomen was performed. A second chemoembolization was performed if the tumor blood supply was not completely blocked. Chemoembolization was performed once in eight cases and twice in seven cases.

### Criteria for evaluation of therapeutic effect

Routine blood tests for liver and kidney function were re-examined at days 3 to 7s after the procedure. Re-examination of hepatic CT was performed at 3 days, 1 month, 3 months, and 6 months after the procedure. The treatment responses were divided into four categories according to the Response Evaluation Criteria in Solid Tumors (RECIST): complete response (CR), partial response (PR), stable disease (SD), and progressive disease (PD), defined as follows. CR: all target lesions disappear and the diameters of all the pathological lymph nodes (including targeted nodes (with a short diameter ≥ 15 mm by CT measurement) and non-targeted nodes) are reduced to less than 10 mm; PR: the sum of all diameters of all target lesions is reduced by 30% from the baseline level; and SD: the degree of reduction does not reach the level of PR and the degree of increase does not reach the level of PD. For PD, there must be at least a 20% increase in the sum of the shortest diameters of all the measured targeted lesions during the whole experimental study (if the value of baseline measurement is the smallest, the baseline value is used as reference). The increase of absolute value of the sum of the diameters should be >5 mm. Furthermore, the appearance of one or more new lesions is also considered as PD. The total response rate equals CR plus PR.

## Results

### Patient characteristics

In total, 15 patients who received GSP-mediated chemoembolization in our department during the period December 2009 to December 2010 were included in this study. The characteristics of the patients are listed in Table 1. The study population consisted of eight men and seven women with a median age of 67 years (range 50 to 80 years). The hepatic function as indicated by Child-Pugh grade was grade A in eleven patients and grade B in four. Diagnoses were determined by needle biopsies, confirming five cases of poorly differentiated adenocarcinoma, nine cases of intermediately differentiated adenocarcinoma, and one case of well-differentiated adenocarcinoma. Based on the CT findings, there were five cases with abdominal lymph node metastases, two with lung metastases, and one with ascites.

**Table 1:** Characteristics of the enrolled patients

**Characteristics**	
Age, years, median (range)	67 (50 to 80)
Gender, n (%)	
Male	8 (53%)
Female	7 (47%)
Hepatic function (Child-Pugh grade), n (%)	
Grade A	11 (73%)
Grade B	4 (27%)
Histology	
Poorly differentiated adenocarcinoma	5 (33%)
Intermediately differentiated adenocarcinoma	9 (60%)
Well-differentiated adenocarcinoma	1 (7%)
Liver lesions, n (%)	
1	5 (33%)
2	2 (13%)
>5	8 (53%)
Metastasis to other organs, n (%)	
No extra-hepatic metastasis	8 (53%)
Lung	2 (13%)
Abdominal lymph node	5 (33%)

### Digital subtraction angiography

Using DSA, three types of tumor vessel characteristics were seen in these 15 patients: 1) The colorectal liver metastases with adequate blood supply showed a similar appearance to primary hepatocellular carcinoma, such as thickened and tortuous hepatic arteries and strong staining of tumor parenchyma; 2) the colorectal liver metastases with reduced blood supply showed ring enhancement in tumors; and 3) the colorectal liver metastases with little blood supply showed rare, thin, stiff or wrapping tumor vessels and edge enhancement of tumor parenchyma. Based on DSA examination, there were eight cases with adequate blood supply (type 1), three cases with reduced blood supply (type 2), and four cases with little blood supply (type 3). DSA examination also showed a single lesion in five cases and multiple lesions in the other ten (Table 1).

### Therapeutic effects

**Figure 1:**
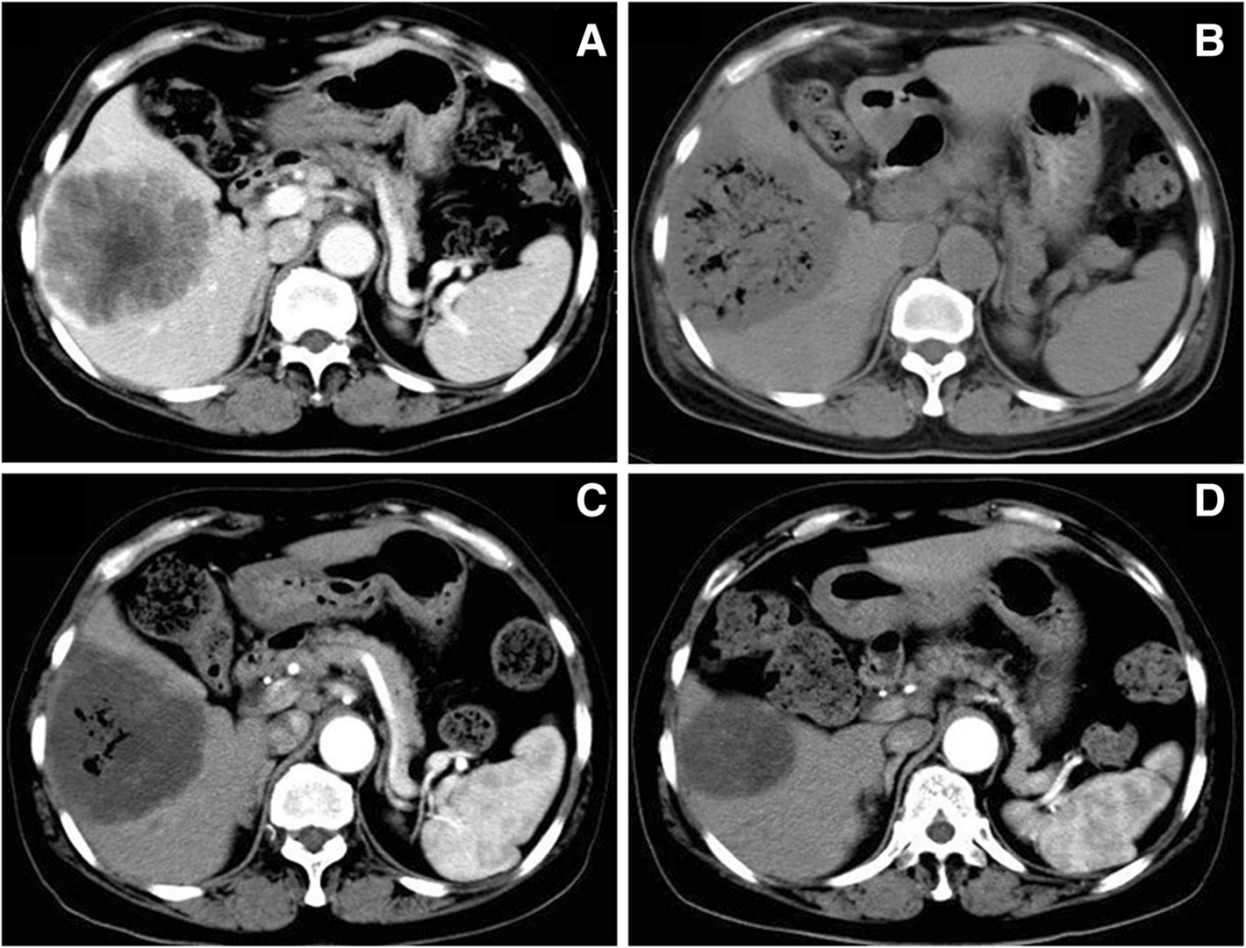
**Alteration of tumor lesions before and after chemoembolization by hepatic CT.** All images are from the same patient. **(A)** Tumors showed ring enhancement before embolization; **(B)** 3 days after embolization, re-examination by CT showed honeycomb-like necrosis of tumor tissue; **(C)** tumors displayed shrinkage 1 month after embolization, without intensification in the lesions; **(D)** 3 months after embolization, more profound tumor shrinkage was visible.

At 3 days, 1 month, and 3 months after the procedure, re-examination of hepatic CT showed that all the tumor lesions exhibited different levels of necrosis and shrinkage (Figure 1A-D). Hepatic function recovered to pre-procedure levels by 7 days after the procedure. At 3 months after the initial procedure, we evaluated the treatment efficacy according to RECIST: patient had CR, eleven patients had PR and three patients had SD. The overall response rate (CR + PR) was 80% (Table 2). At 6 months after the procedure without any other therapy, additional hepatic CT showed that twelve patients had PR, one patient had SD, and two patients had PD. No deaths occurred.

**Table 2:** Therapeutic effects 3 months after the procedure

**Patient number**	**Age, years**	**Gender**	**Pathological types**	**Liver lesions, b**	**Metastasis to other organs before chemoembolization**	**Embolizations, n**	**Effect**
1	80	Male	Intermediately differentiated	2	No	1	PR
2	72	Female	Poorly differentiated	1	No	2	PR
3	67	Female	Intermediately differentiated	1	Lung	2	SD
4	58	Male	Intermediately differentiated	> 5	No	1	PR
5	60	Female	Intermediately differentiated	2	No	2	CR
6	56	Male	Intermediately differentiated	1	No	1	PR
7	73	Female	Poorly differentiated	> 5	Abdominal lymph node	2	PR
8	54	Male	Poorly differentiated	> 5	Lung	2	PR
9	50	Female	Poorly differentiated	> 5	Abdominal lymph node	1	SD
10	78	Male	Intermediately differentiated	> 5	Abdominal lymph node	2	PR
11	68	Male	Intermediately differentiated	> 5	No	1	PR
12	64	Male	Well differentiated	1	No	1	PR
13	69	Female	Intermediately differentiated	1	Abdominal lymph node	2	PR
14	68	Male	Intermediately differentiated	> 5	Abdominal lymph node	1	PR
15	65	Female	Poorly differentiated	> 5	No	1	SD

CR, complete response; PR, partial response, SD: stable disease.

### Side effects

Treatment-related side effects in the 15 patients enrolled in the study included fever, mild stomach discomfort, nausea, and vomiting. Conservative treatment for these symptoms resolved side effects within 7 days. No serious complications such as liver and kidney failure, liver abscess, or biloma were seen.

## Discussion

Owing to the biological characteristics of the tumors and the anatomic characteristics of the colorectum, patients with CRC have a tendency to develop liver metastases [14], and liver metastasis accounts for 60 to 71% of CRC deaths [15]. Surgical resection is still the primary therapeutic method for prolonging the survival of patients with colorectal liver metastases, but unfortunately, 70 to 85% of patients are deemed unresectable when evaluated [16]. For patients with unresectable liver-dominant disease, TACE is an efficient therapeutic method [10,11,17,18]. Hepatic arteries supply 95% of the blood flow to liver neoplasms, whereas 25% of the blood flow of the normal liver is supplied by hepatic arteries and 75% is supplied by the portal vein [19,20]. This anatomical feature allows a high concentration of chemotherapeutics to directly act on tumor cells through hepatic arteries, and appropriate embolization agents can be chosen to block blood supply of tumors. This therapy has been shown to improve control of hepatic metastases and further improve quality of life and survival [21,22].

Arterial embolization agents are currently divided into two categories: permanent and absorbable. Lipiodol is a commonly used permanent embolization agent. It has a superior therapeutic effect for solid tumors with adequate blood supply, but has an inferior therapeutic effect for those with poor blood supply. Furthermore, it results in severe liver and kidney damage due to its mass effect [23], and cannot perform complete embolization for large-volume liver tumors [24,25]. Recently, the use of microparticles as embolization agents has attracted increased attention. Several clinical reports have shown that drug-loaded microspheres can achieve superior effects in embolization of liver metastases [26-28]. Nitta *et al*. reported that single embolization therapy performed through the hepatic arteries in tumor regions in nine cases with liver metastases, using cisplatin-releasing gelatin microspheres of 50 to 100 μm in size, resulted in two cases of CR, one case of PR, and six cases of SD [29]. Notably, all the embolization agents used above were permanent embolization agents, with which complete embolization for large-volume liver tumors cannot be achieved, and a risk of liver failure, gallbladder necrosis, liver abscesses, and other serious complications exists [12,30].

GSPs are effective absorbable embolization agents that have been used in clinical practice [12]. The GSP we used in this study have an absorption time of 7 to 14 days and increased homogeneity, and therefore cause less damage to hepatic function and can be used to perform complete embolization of the tumor-feeding arteries. In our study, CT scans showed honeycomb-like necrosis throughout the tumor area 3 days after the procedure, and the hepatic function recovered to pre-procedure levels by 7 days. At 1 month after the procedure, the tumors showed various levels of low-density necrosis and shrinkage, and were effectively controlled.

## Conclusion

Application of GSPs as an embolization agent combined with 50 mg of Lobaplatin can completely block the arteries of metastatic liver lesions and achieve a high tumor response rate (80%). Other advantages include no mass effect, reduced liver damage, and rapid recovery of post-procedure hepatic function. GSP-mediated chemoembolization achieved good short-term effects in the treatment of colorectal liver metastases. However, the long-term effects remain to be elucidated and studies with larger sample size are warranted.

## Abbreviations

CRC: Colorectal cancer; GSP: Gelatin sponge particle; RECIST: Response Evaluation Criteria in Solid Tumors; CR: Complete response; PR: Partial response; SD: Stable disease; PD: Progressive disease; TACE: Transcatheter arterial chemoembolization; 5-FU: 5- fluorouracil; FOLFOX: 5-FU/leucovorin/oxaliplatin; FOLFIRI: 5-FU/leucovorin/irinotecan; CFDA: China Food and Drug Administration; CT: Computed tomography; DSA: Digital subtraction angiography.

## Competing interests

The authors declare that they have no competing interests.

## Authors’ contributions

CL and GZ participated in the design of the study, performed the statistical analysis, and drafted the manuscript. JZ and ST participated in study design and literature search. YZ conceived of the study, participated in the design, and helped to draft the manuscript. All authors read and approved the final manuscript.
